# Investigation of the Effect of Mixing Time on the Mechanical Properties of Alkali-Activated Cement Mixed with Fly Ash and Slag

**DOI:** 10.3390/ma14092301

**Published:** 2021-04-29

**Authors:** Taewan Kim, Choonghyun Kang

**Affiliations:** 1 Department of Civil Engineering, Pusan National University, Busan 46241, Korea; ring2014@naver.com; 2 Department of Ocean Civil Engineering, Gyeongsang National University, Tongyeong 53064, Korea

**Keywords:** slag, fly ash, mixing method, high alkali environment, mixing time

## Abstract

This is an experiment on the effect of mixing time for alkali-activated cement (AAC) using a binder mixed with ground granulated blast furnace slag (slag) and fly ash (FA) in a ratio of 1:1 on the mechanical properties. The mixing method of ASTM C305 was used as the basic mixing method, and the following mixing method was changed. Simply adding the same mixing time and procedure, the difference in the order of mixing slag and FA, and controlling the amount of activator and mixed water were considered. As a result of the experiment, the addition of the same mixing time and procedure, pre-injection of slag, and high-alkali mixed water in which half of the activator and mixing water were mixed showed the highest mechanical properties and a dense pore structure. As a result, the design of a blending method that can promote the activation action of slag rather than FA at room temperature was effective in improving the mechanical properties of AAC. In addition, these blending factors showed a clearer effect as the concentration of the activator increased. Through the results of this experiment, it was shown that high-temperature curing, high fineness of the binder, or even changing the setting of the mixing method without the use of excessive activators can lead to an improvement of mechanical properties.

## 1. Introduction

Alkali-activated cement (AAC) has attracted considerable attention as a next-generation cement that could replace existing cement materials, and many researchers are currently conducting research on AAC [1]. The ground granulated blast furnace slag (slag) and fly ash (FA) are binary binders used in AAC, and many AAC studies using these binders have been published [2,3].

In general, the higher the mixing ratio of slag in a slag–FA mixed binder, the higher the strength of the concrete [4,5,6,7,8,9,10], because the reaction of FA at ambient temperature is considerably low [4,5,11,12,13,14,15,16]. Therefore, at ambient temperature, FA-based geopolymers exhibit long setting times and low strength [4,11,17,18,19]. However, at curing temperatures above 60 °C, FA-based geopolymers exhibit fast setting times and high strength [14,20,21,22,23,24,25,26,27]. Therefore, mixing FA and slag at high temperature can result in accelerated setting and enhanced strength [15,18,28,29,30,31].

There are many factors to consider when mixing slag and FA. Factors considered in previous studies include various slag/FA mixing ratios [4,15,32,33,34], curing temperatures (25 °C and 65 °C [4], 50–80 °C [22], 22 °C and 65 °C [35], 95 °C [36], 65 °C, 70 °C, and 80 °C [37], 27 °C and 60 °C [15], and 20 °C and 70 °C [38]), types of activators (sodium hydroxide [4,32,39], sodium silicate [36], sodium metasilicate [29], sodium silicate and sodium hydroxide [15,40,41,42], liquid and powder sodium silicate [7], sodium hydroxide, sodium silicate, and sodium hydroxide and sodium silicate [8,19,28], sodium carbonate [43,44], sodium carbonate and sodium silicate [45], and potassium silicate [31]), activator concentrations [34,40,46], slag grades [5], slag nature [47,48,49], types and amounts of superplasticizers [6,50], water-to-binder ratio [51,52], and admixtures (nano-silica [53] and limestone [54]). These various influencing factors have led to the study of the properties of AAC using slag-FA-mixed binders [2,52,55].

The main hydration reaction product of AAC using slag as the sole binder is a calcium silicate hydrate (C-S-H) gel, and the main hydration reaction product of AAC using FA as the sole binder is an aluminosilicate (N-A-S-H) gel [4,35,56,57]. AAC, a mixture of slag and FA, has a more complex hydration mechanism than when each is used alone. The slag-FA mixed specimens are composed of a calcium (aluminum) silicate hydrate (C-(A)-S-H) gel and a sodium aluminosilicate hydrate (N-A-S-H) gel, which form a compact microstructure [4,15,18,29,31,32,58,59]. In addition, a hydrotalcite-like phase, pirssonite, and calcite have been observed [4]. To date, various studies on slag-FA based alkali-activated cement have been reported, mainly referring to the improvement of the mechanical properties when the ratio of slag is increased or the curing temperature is high.

The aim of this experiment is to investigate the characteristics of binder materials with sodium hydroxide and sodium silicate as an activator and slag and FA in a weight ratio of 50:50. A total of four methods were considered. Each method was designed differently according to addition time and sequence, division of mixing water, slag, or FA injection order. The characteristics of compressive strength, hydration reactant, and pore structure were analyzed according to each mixing method.

## 2. Materials and Experiments 

### 2.1. Materials

The properties of slag and FA used in this study are summarized in Table 1 as obtained via an X-ray fluorescence analysis (XRF-1800, Shimadzu, Kyoto, Japan). The binder was prepared by mixing slag and fly ash at a weight ratio of 50:50, respectively. The alkali activator used in the study was NaOH (purity ≥ 98%, pellet type) and Na_2_SiO_3_ (Ms = 2.0, liquid type).

The dosage of activator was 5% and 10%. The 5% activator means NaOH and Na_2_SiO_3_ (5% NaOH + 5% Na_2_SiO_3_) corresponding to 5% of the binder weight, and the 10% activator means 10% NaOH + 10% Na_2_SiO_3_. Immediately prior to the start of the experiment, 5% and 10% activator was added to the mixing water, and the mixture was well stirred and placed in the laboratory for about 6 h.

### 2.2. Experiments

The water-to-binder ratio (w/b) of the paste was 0.5, the mixing time and procedure were based on ASTM C305 [60], and the mixing time was adjusted to form a homogeneous slag-FA mixture. After the materials are mixed in a mixer, the pastes were put in to the mold and stored in the chamber (relative humidity 90% ± 5% and 23 °C ± 2 °C) for one day. Then, the mold was demolded, and the samples were stored in the chamber (relative humidity 90% ± 5% and 23 °C ± 2 °C).

The procedures for mixing the pastes for each method are as follows:

- The control mixing procedure and time were based on ASTM C305. The orders for the procedures were the same; however, the stop time, which is the next level of slow speed, has been increased from 15 to 30 s. This is to ensure sufficient time required for the scraping of the paste. The description of the mixing method designed in this study is as follows.
- Method-S mixed slag and FA with the pre-mixed alkali activator for 30 s at a low speed (140 ± 5 r/min), after which the process was stopped for 30 s while the paste attached to the wall of the mixing bowl was scraped down. A second 30 s round of mixing took place at a medium speed (285 ± 10 r/min), followed by a 90 s stop, during which the bowl was scraped again. Then, a third round of mixing was performed for 60 s at the medium speed. In total, the mixing took 270 s.
- Method-A followed the same mixing procedure as Method-S; and after the final mixing step is over, repeat the entire mixing time and sequence once more. At this time, there is no addition of additional materials in the intervertebral mixing time and sequence.
- Method-B placed slag in the liquid solution containing half the mixing water and the activator as used in Method-S and then performed the first mixing cycle. Then, this mixture was combined with FA and half of the mixing water (without the alkali activator), and then, the second mixing cycle was performed.
- Method-C was formed in the same manner as Method-B except that the FA was added in the first cycle and the slag was added in the second cycle. Since both Method-B and Method-C consisted of two cycles, it took 540 s to finish the mixing for these methods.

The methods can be summarized as the basic mixing method (Method-S), simply adding a mixing procedure (Method-A), mixing the slag first (Method-B), and mixing the FA first (Method-C).

The process and time for the mixing methods are summarized in Table 2.

A compressive strength test was performed in accordance with ASTM C109 [61] using a specimen size of 50 × 50 × 50 mm^3^. The loading rate was 1600 N/s, and the compressive strengths were carried out on three specimens at 1, 3, 7, and 28 days, and their average values were used.

X-ray diffraction (XRD) analyzes were carried out at 1 and 28-day samples to identify hydration reactants. The sample for XRD analysis was prepared by immersing the crushed sample fragments after the compression strength measurement in acetone for 12 h, stopping hydration, drying in a vacuum-chamber for 24 h, and then pulverizing the crushed sample. The XRD was measured with XPert-3 from PANalytical (PANalytical, Almelo, The Netherlands). The measurement conditions are Cu K-α is 1.540598, measurement range from 5 to 60° (2θ), scan step of 0.013° (2θ), 40 kV, and 30 mA. Analysis of pore structure was carried out at 1 and 28-day specimens with the mercury intrusion porosimetry (MIP). MIP was measured using an AutoPore IV (Micromeritics, Norcross, GA, USA). Specimens for MIP measurement were cut into a size of 5 × 5 × 5 mm^3^ for the central part of three 50 × 50 × 50 mm^3^ samples using a precision cutter. The contact angle is 130.0°, the surface tension of mercury (Hg) is 485.0 dynes/cm, and the density of Hg is 13.5335 g/mL. Thermogravimetric/differential thermal analysis (TG/DTA) analyses were performed at 1 and 28-day-aged samples using a DSC800 thermal analyzer (Perkin Elmer, Waltham, MA, USA) and were measured at 30 to 850 °C in increments of 20 °C/min in a nitrogen gas environment.

## 3. Results and Discussions

### 3.1. Compressive Strength

**Figure 1:**
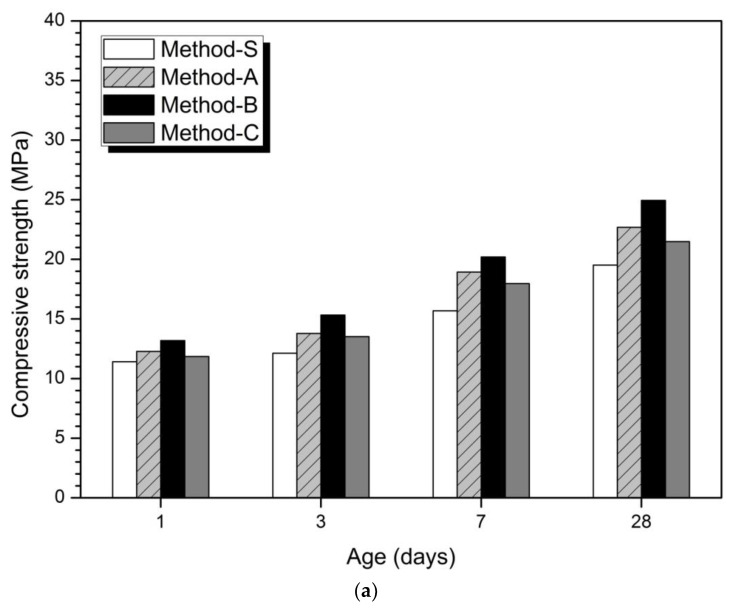

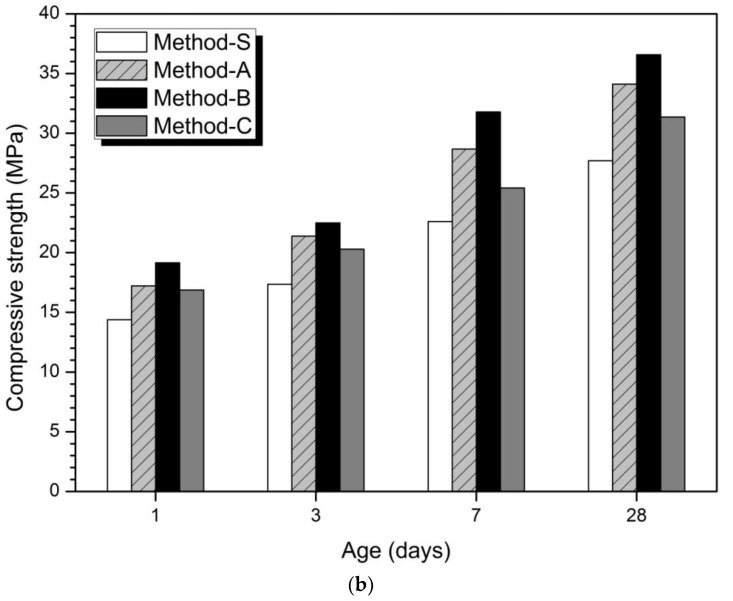
Compressive strength according to concentration and mixing method of activator (**a**) 5% activator, (**b**) 10% activator.

Figure 1 depicts the results of compressive strength measurement results according to the mixing method. In the 5% activator of Figure 1a and the 10% activator of Figure 1b, Method-S showed the maximum compressive strength and Method-S showed the minimum value. The order of compression strength magnitude is Method-B > Method-A > Method-C > Method-S. These compressive strength magnitudes were confirmed at all measurement ages. From the strength results in Figure 1, it can be seen that just changing the mixing method can affect the mechanical properties. This means that the compressive strength can be improved without increasing the dosage of the activator, decreasing the w/b ratio, and increasing the curing temperature.

Previous studies have reported that the ratios of slag affect the strength AAC or geopolymer blends of slag and FA [4,6,7,30,31,34,62,63,64,65]. The reason why the amount of slag affects the increase of compressive strength is that slag is more reactive in alkaline environment than FA and produces a more dense hydration reaction [15,47]. 

However, in the case of a binder with a constant composition ratio or amount of slag and FA, the strength varies depending on various factors—for example, the nature of slag and FA, type and concentration of activator, added admixture, superplasticizer, and curing temperature. If all the conditions are the same, a new method for improving the strength of the slag–FA binder can be expected to help improve the mechanical properties.

Table 3 shows the percentage increase in relative compressive strength for each method compared to Method-S. The highest compressive strength growth rates at 1, 3, 7, and 28 days are observed in Method-B. In the 5% activator and 10% activator at 1 day, the compressive strength increase rate of Method-B was the highest, followed by Method-A and Method-C. Table 3 shows that the mixing method affected the initial hydration stage and resulted in an increase in the initial strength at 1 day. Method-B showed the highest compressive strength growth rate at all measurement days of 1, 3, 7, and 28 days. Therefore, the mixing method was found to have a continuous effect until 28 days after 1 day.

The mixing method considered in this study is different in the concentration of activator and the order of introduction of slag-FA. Method-B, where the maximum compressive strength was measured, produced a high dosage of alkali solution by mixing the activator and half of the mixed water in the first cycle. Slag is added to the prepared solution and mixed. Then, half of the mixture and the FA are added to the second cycle to mix. Therefore, Method-B accelerates the hydration of slag by establishing a higher alkaline environment than Method-A, which mixes the total mixing water with the activator in the first cycle. The higher the concentration of the activator, the better the hydration reactivity of the slag, and as a result, the effect of improving the compressive strength is promoted [33,34,66]. As a result, Method-A exhibits a similar effect of diluting the activator concentration more than Method-B, resulting in a slight decrease in strength. Reduction of the activator concentration reduces the hydration of slag-FA and reduces the initial compressive strength or the overall compressive strength value [67,68]. Method-C is also the same high-alkaline environment as Method-B, but we put FA into the first cycle. Slag was put into the second cycle.

Previous studies to date have reported that at room temperature, FA has a lower hydration reaction than slag [4,5,11,15,17,18,19]. High-concentration alkali concentration and high curing temperature are required for FA hydration [14,20,21,22,23,24,25,26,27]. In this high-temperature curing environment, the strength improvement effect was also observed in the binder mixed with slag and FA [18,28,29,30,31]. Therefore, under the conditions of this study, FA has a lower hydration reactivity than slag, so that the method of injecting FA into the first cycle is not effective in improving the compressive strength. As a result, Method-C is a high-alkaline environment similar to Method-B, but the effect of improving the compressive strength is low due to the method of injecting FA first.

From the results of compressive strength, the strength improvement effect could be obtained by simply changing the amount of activator and mixed water and the order of addition of slag and FA.

### 3.2. Hydration Products

**Figure 2:**
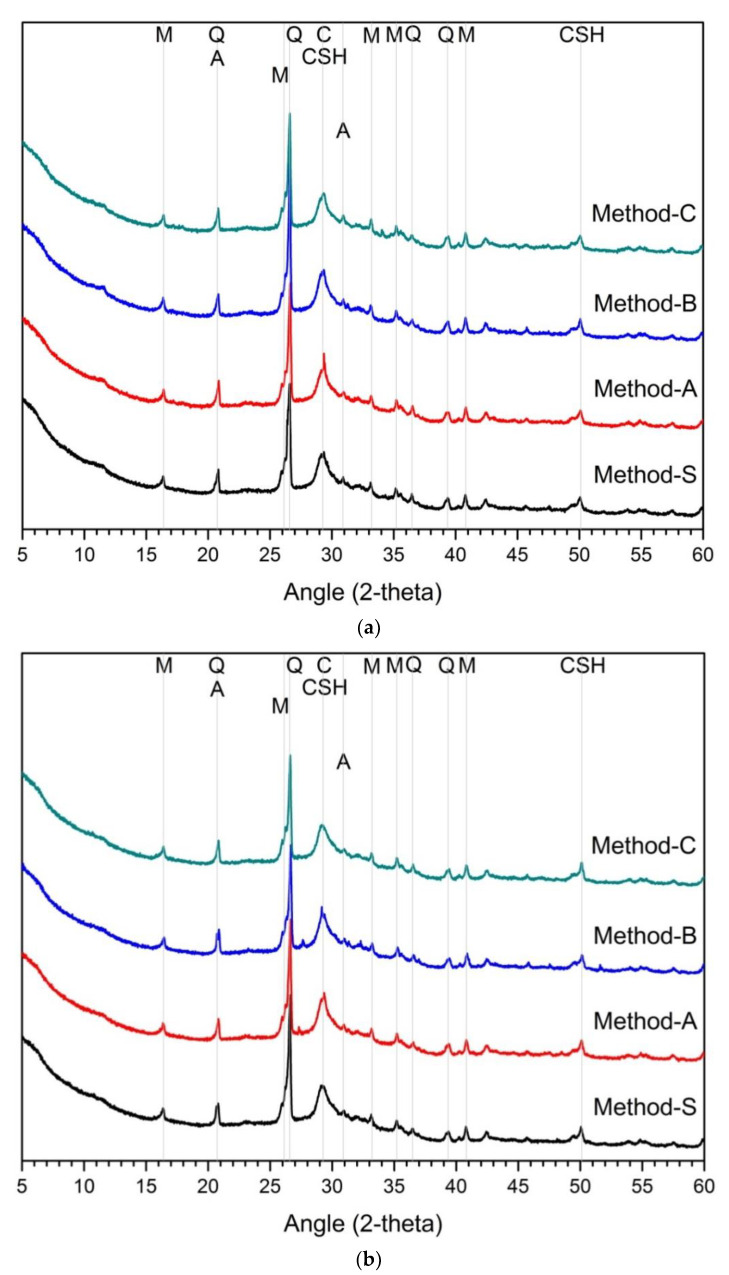

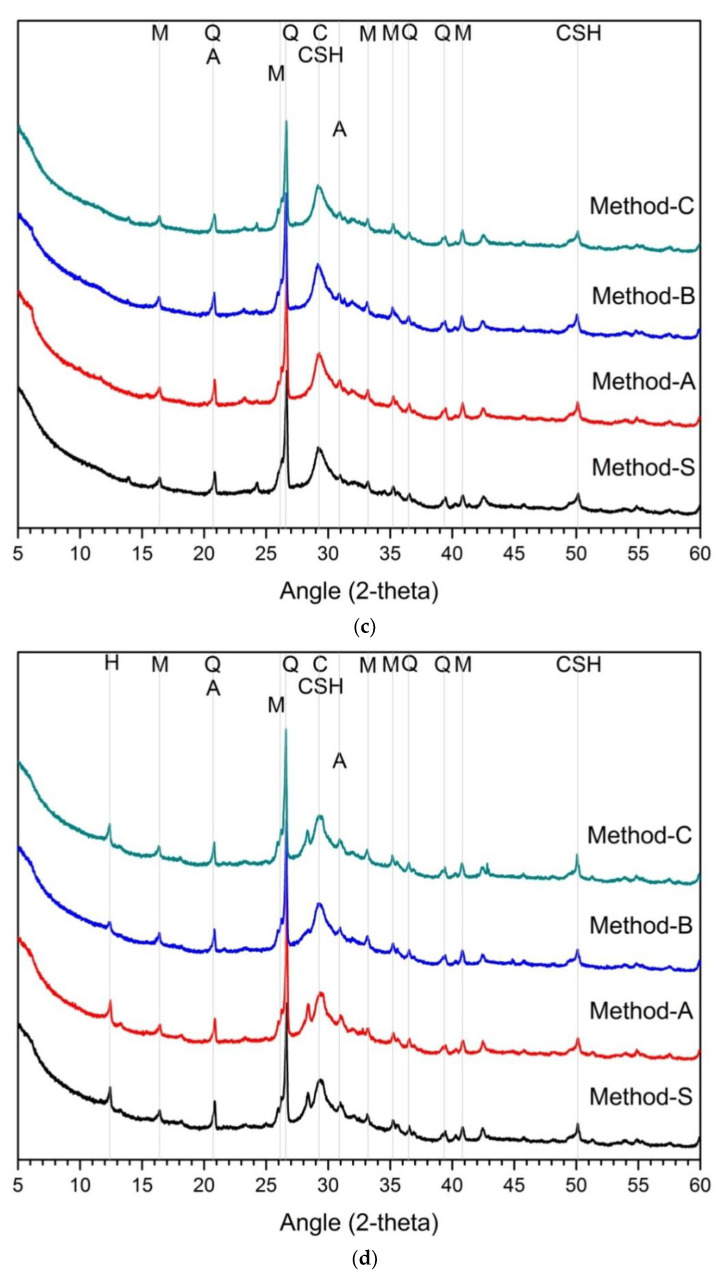
XRD analysis results on hydration reactants (**a**) 5% activator, 1 day, (**b**) 5% activator, 28 days, (**c**) 10% activator, 1 day, (**d**) 10% activator, 28 days, Q: quartz, H: hydrotalcite, M: mullite, C: calcite, A: akermanite, CSH: C-S-H gel.

Figure 2 is the analysis result of hydration products according to the mixing method. The main hydration products are C-(A)-S-H gel and calcite. Several research reports have reported that the main hydration products of alkali-activated cement mixed with slag and fly ash is C-A-S-H gel [19]. In the XRD analysis, akermanite of raw-slag and mullite of raw-FA were observed irrespective of the concentration of active agent and mixing method. The hydration products at 1 day and 28 days for 5% and 10% activators were quite similar. There was no difference in the hydration products according to the mixing method, and no new hydration products were observed. Therefore, according to the analytical results of hydration products, the difference of mixing method has little effects on the change of hydration product.

In the case of slag–FA binder, the higher the substitution ratio of slag, the more the hydration products are increased [7,62,69]. This is because FA reactivity at room temperature is low [5,28,59,63,70]. As a result, at room temperature, the hydration reactivity of slag affects the formation and amount of hydration products. Therefore, in previous studies, slag showed higher activation effect than FA in the study of alkali-activated cement or geopolymer of slag-FA binder [15,18,47]. However, the effect of the blended ratio of slag and FA was not considered in this study. That is, in the experiment, only the blended ratio of slag and FA was designed to be 1:1. For the hydration reactant, only the influence of the mixing method is considered, not the mixing ratio of slag and FA. As a result, the change and difference of the hydration reactant in Figure 2 are only due to the mixing method. Unlike the compressive strength, the change of the hydration products was almost insignificant, despite the differences in the mixing methods such as the concentration of the activator in the first cycle and the order of addition of slag or FA.

It can be estimated that the mixing method does not affect changes in hydration products. It is not enough to explain the effect on the fluctuation of compressive strength because the change of hydration products according to the mixing method is insignificant. Therefore, it can be considered that there are other factors that can explain the change in mechanical performance.

### 3.3. Pore Structures

**Figure 3:**
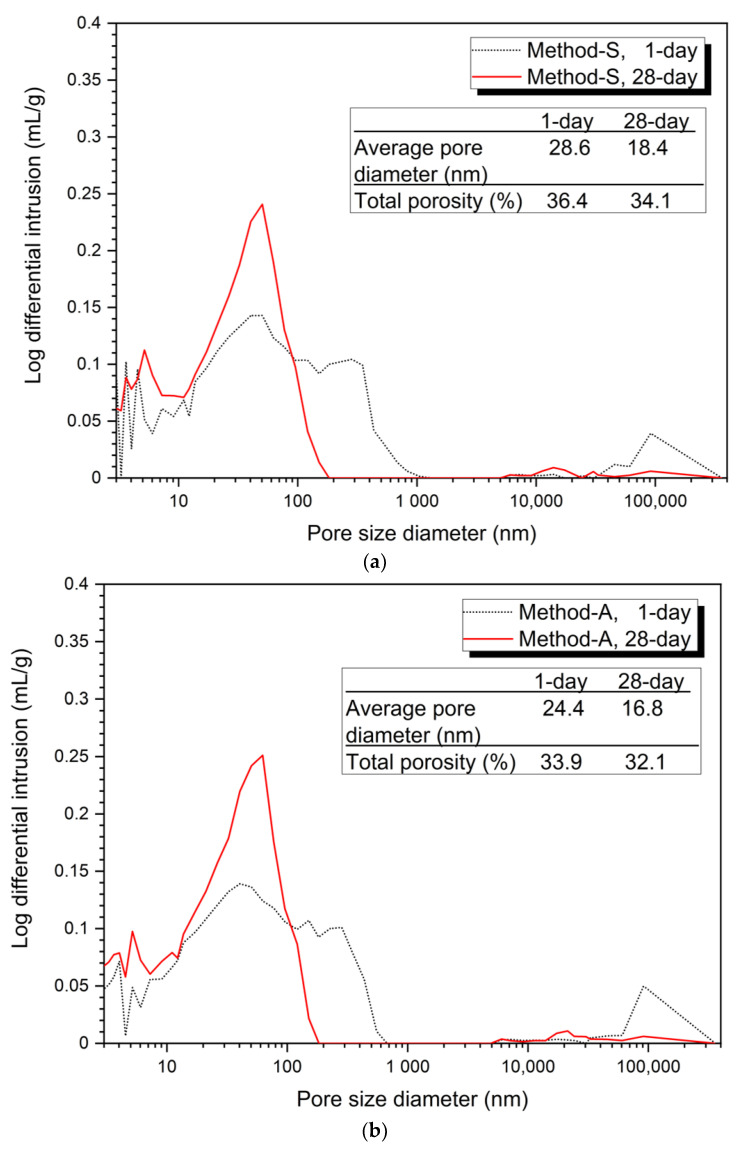

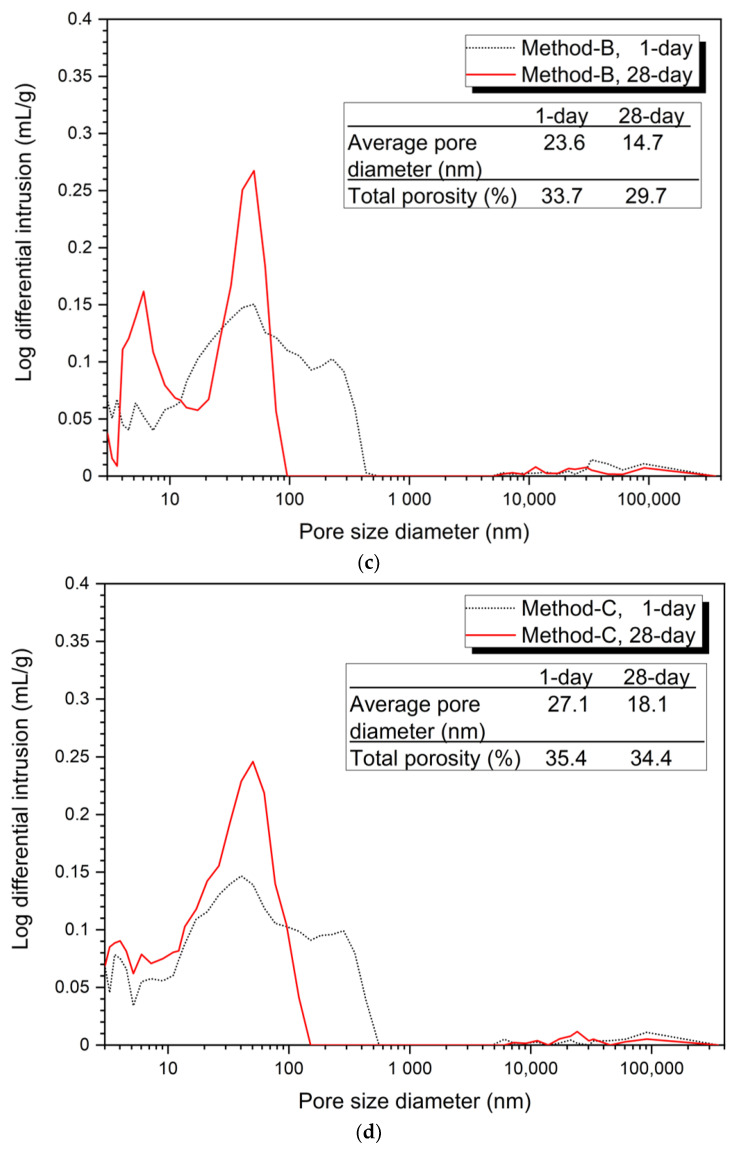

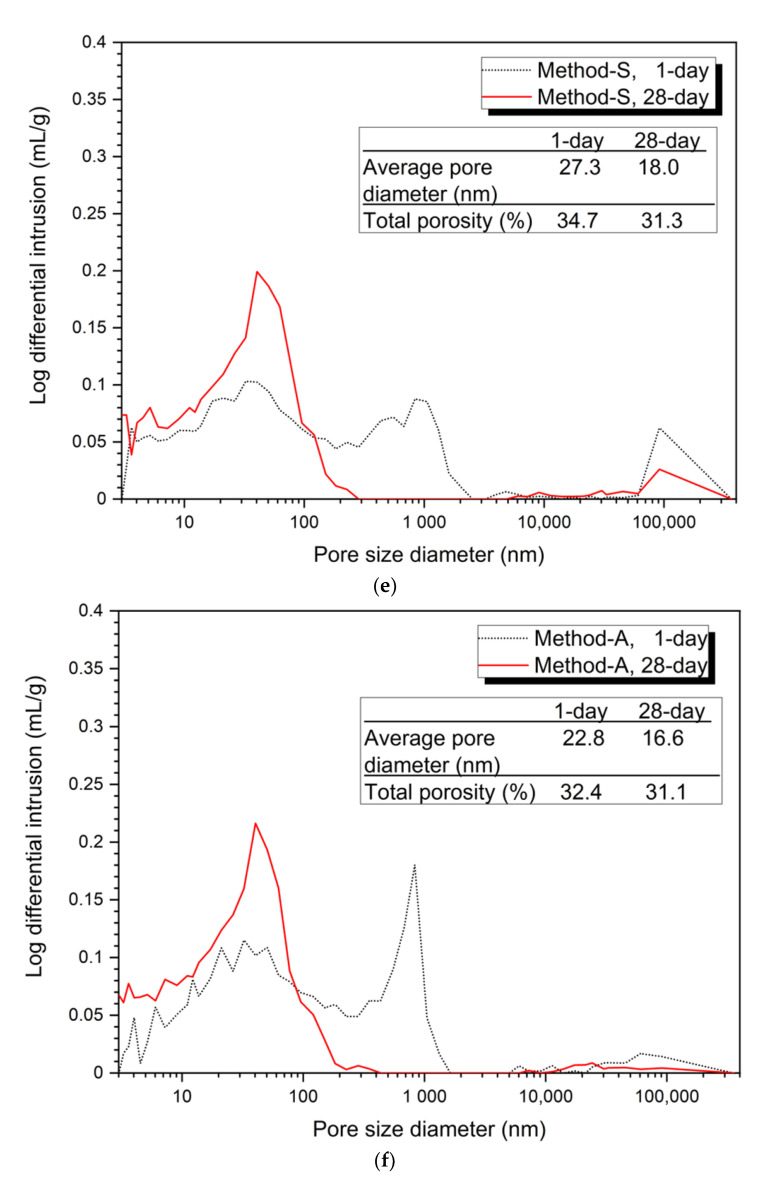

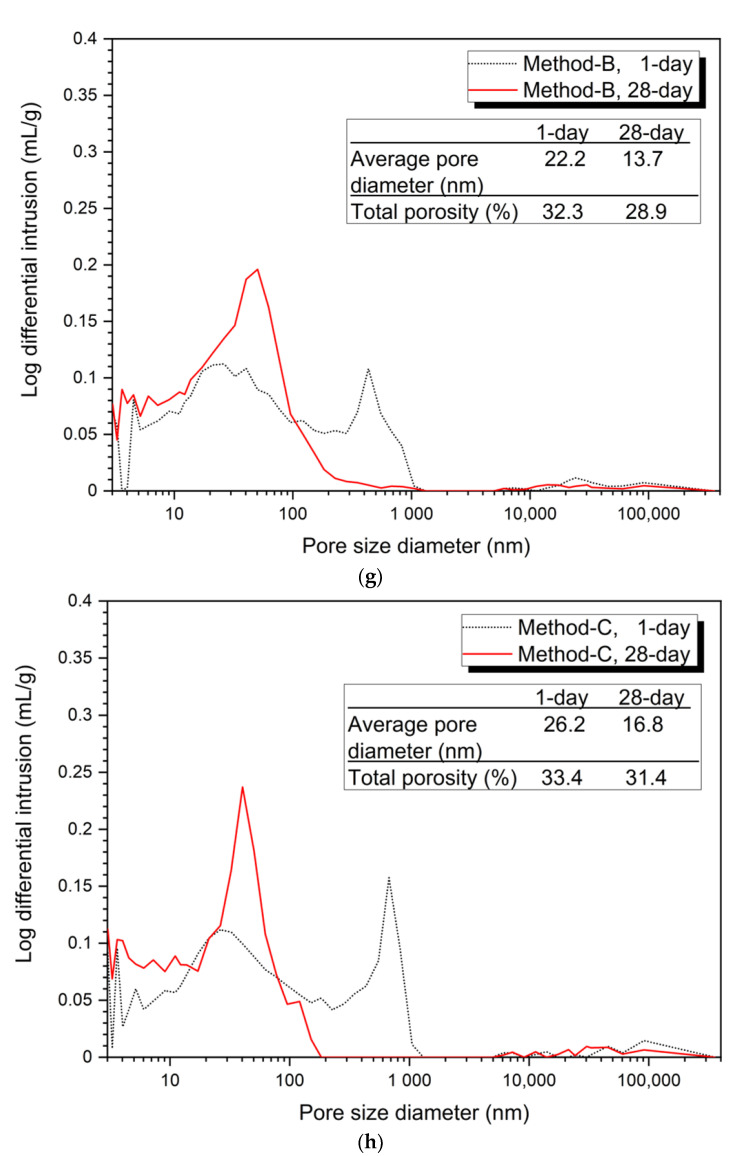
Pore distribution by mixing method, (**a**) 5% activator, Method-S, (**b**) 5% activator, Method-A, (**c**) 5% activator, Method-B, (**d**) 5% activator, Method-C, (**e**) 10% activator, Method-S, (**f**) 10% activator, Method-A, (**g**) 10% activator, Method-B, (**h**) 10% activator, Method-C.

Figure 3 shows the analysis of the pore structure according to the mixing method. Bothe 5% and 10% activator samples, the size and volume of pores at 28 days were reduced in all mixing methods compared to at 1 day. This can be clearly seen from the average pore diameter and total porosity values at 1 day and at 28 days.

Figure 3a–d show the porosity distribution of the 5% activator and Figure 3e–h show the porosity structure for the 10% activator. Figure 3 shows that the tendency of the variation of the average pore diameter and total porosity according to the mixing method of 5% and 10% activator samples is similar. 

The 1-day average pore diameter of the 5% activator was the smallest at 23.6 nm for Method-B and gradually increased to 24.4 nm for Method-A, 27.1 nm for Method-C, and 28.6 nm for Method-S. Total porosity increased in the order of 33.7% (Method-B), 33.9% (Method-A), 35.4% (Method-C), and 36.4% (Method-S). The average pore diameter was increased in the order of the method-B (14.7 nm) < Method-A (16.8 nm) < Method-C (18.1 nm) < Method-S (18.4 nm). The total porosity increased by 29.7% (Method-B), 32.1% (Method-A), 34.1% (Method-S), and 34.4% (Method-C). Unlike the average pore diameter, the total porosity of Method-S was 0.3% smaller than that of Method-A.

The 10% activator showed a similar average pore diameter and total porosity as the 5% activator. The average pore diameter values at 1 day and 28 days were found to be Method-B (22.2 nm, 13.7 nm), Method-A (22.8 nm, 16.6 nm), Method-C (26.2 nm, 16.8 nm), and Method-S (27.3 nm, 18.0 nm). The total porosity at 1 day increases to Method-B (32.3%), Method-A (32.4%), Method-C (33.4%), and Method-S (34.7%). However, at 28 days, it gradually increased to Method-B (28.9%), Method-A (31.1%), Method-S (31.3%), and Method-C (31.4%). Unlike the total porosity at 1 day, Method-A, Method-S, and Method-C at 28 days showed very small differences in total porosity between each mixing method.

From the pore structure analysis of Figure 3, we can confirm that Method-B has the smallest average pore diameter and total porosity at 1 and 28 days. The change in pore structure (decrease in average pore diameter and total porosity) can explain the increase in compressive strength in the absence of changes in the hydration products shown in Figure 2. In other words, it is shown that the difference of the mixing method forms a dense matrix, thereby reducing the size and quantity of pores, which affects the improvement of compressive strength.

**Figure 4:**
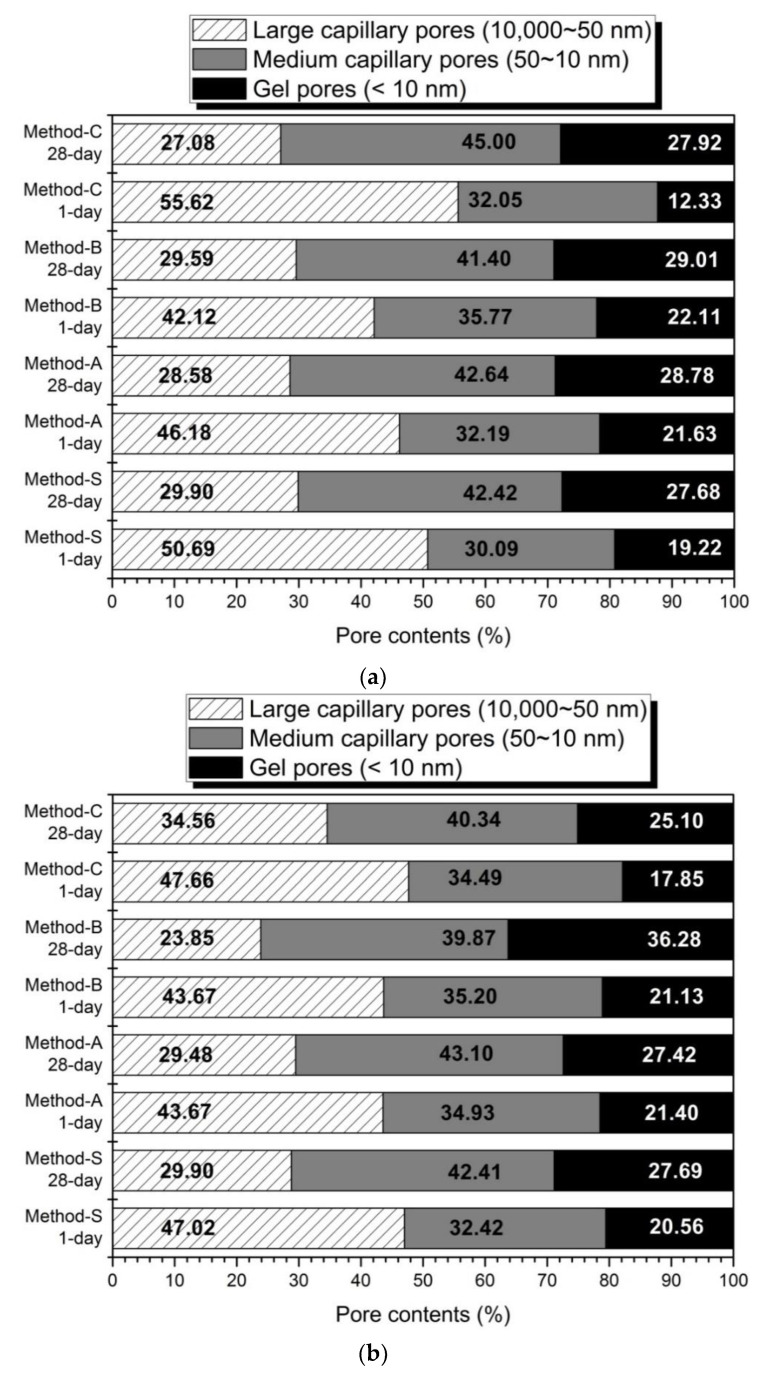
The pores volume ratios of the pore size (**a**) 5% activator, (**b**) 10% activator.

Figure 4 shows the volume ratios according to the pore size from the MIP results of each mixing method. This is indicated by the pore classification of Mindess et al. [71]. In Figure 4, regardless of the activator concentration, the 28-day sample showed a decrease in the large capillary pores compared to the 1-day sample, but the medium capillary pores and gel pores were increased. 

The amount of gel pores in Method-B is higher than the remaining mixing method. The increase in gel pore means that the amount of hydration reactant such as C-S-H gel or C-(A)-S-H is increased [71]. It has been reported that C-(A)-S-H gel, an AAC hydration reagent of slag-base, provides greater pores filling capacity than FA-base geopolymer-type gels [29]. Therefore, hydration enhancement of slag reduces the large capillary pore and increases the medium capillary and gel pores by pores-filling action. As a result, the formation of a dense matrix reduces the total pore reduction and average pore diameter, as mentioned in Figure 3. It also affects the improvement of the compressive strength, as shown in Figure 1.

In slag–FA AAC, an increase in the content of slag or an increase in activator is known to promote the hydration of slag, thereby increasing the contents of pores with a diameter of 50 nm or less and reducing the total porosity [70]. From slag-FA-based AAC studies, it has been reported that slag forms a denser matrix than FA and plays a role in increasing the micro-pore size. [57,72]. The increase of the activation reaction of slag produces a dense hydration reactant to make the matrix more compact, and as a result, it improves the compressive strength [73]. In general, geopolymer gel, a hydration product of FA, has been reported to have less space-filling effect than C-(A)-S-H gel formed by the activation of slag. Therefore, as the content of slag increases in slag-FA AAC, the C-(A)-S-H gel also increases, and the pore size and pore volume decrease [69,74]. The glassy phases of the slag are more vulnerable to alkaline attacks than the aluminosilicate-enriched ones from FA under room temperature [18,25]. In addition, the slag generally has a higher content of reactive phases than does FA [20,71]; therefore, a higher amount of Si and Ca will dissolve, and more hydrated gels will be formed with a slag than with FA [34], which can explain the increase in the compressive strength and the decrease in the porosity with Method B.

In this experiment, the blended ratio of slag and FA is 1:1 by mass ratio. Therefore, the effect of changing the blended ratio of slag or FA is not considered. However, the effect of reducing the pore size and volume was also confirmed by the method of enhancing the formation of C-(A)-S-H gel by accelerating the hydration of slag by forming a high pH environment in the early hydration stage. From the results of this research, the mixing method changes the pore structure. In particular, regarding methods that maximize the activation of slag in high-alkaline environments, Method-B affects the formation of dense matrices. Therefore, the improvement of the compressive strength by the mixing method is more affected by the change of the void structure than by the change of the hydration products of Figure 2.

### 3.4. Thermal Analysis

**Figure 5:**
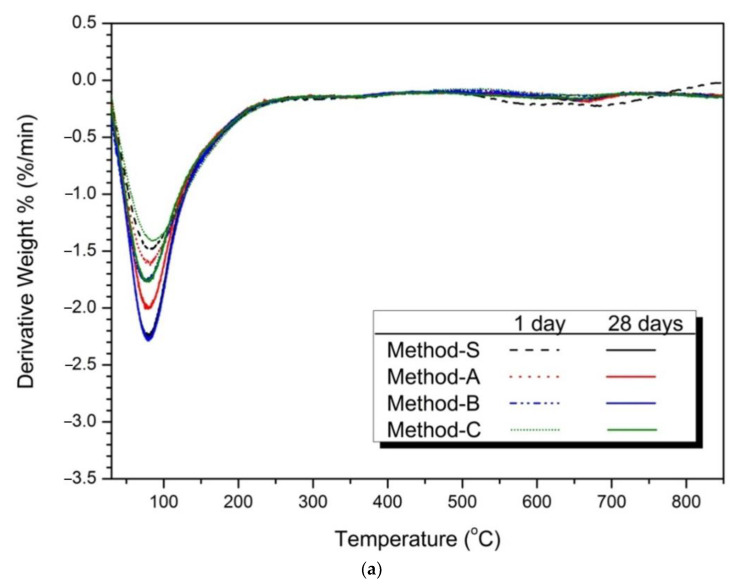

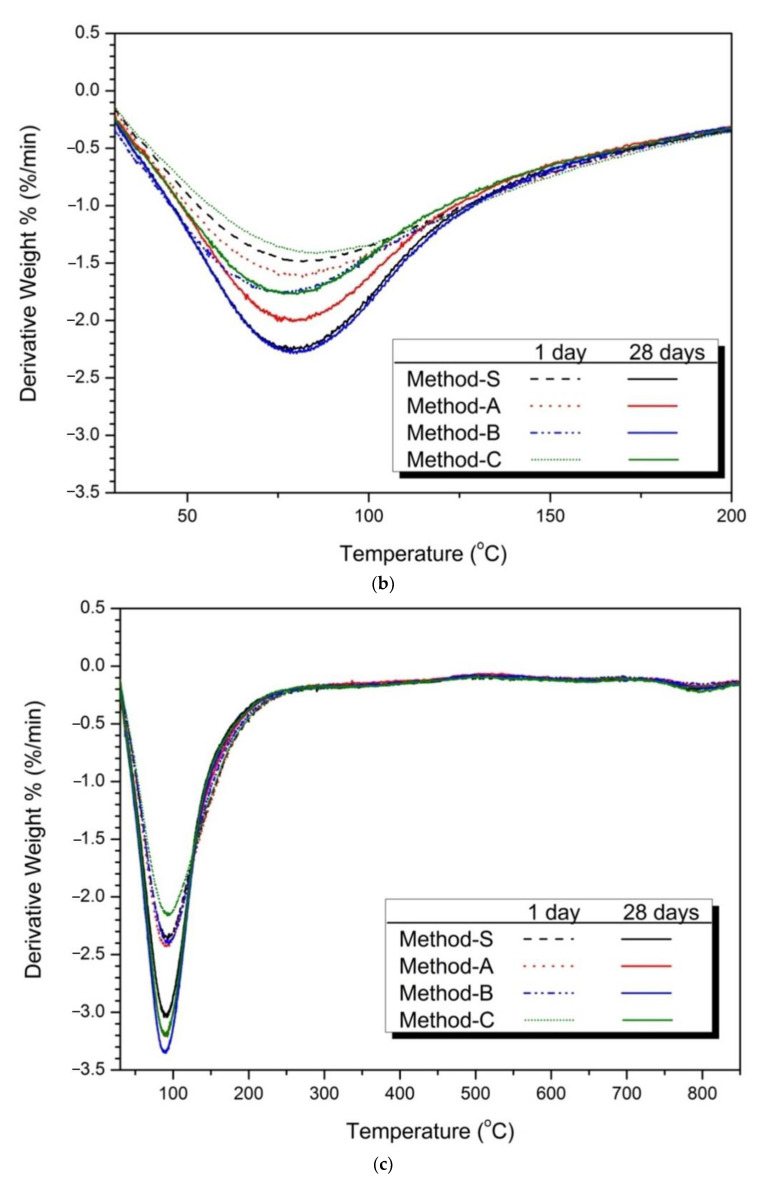

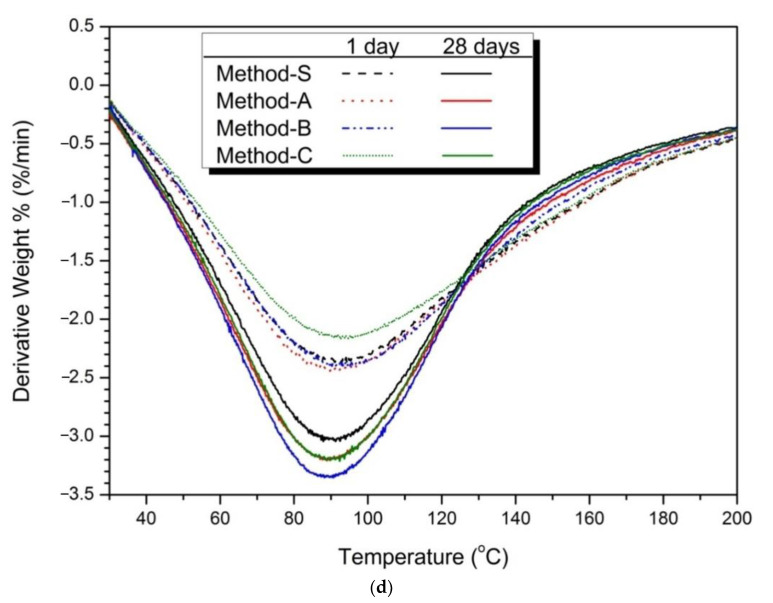
Thermal analysis (TG/DTA results) (**a**) 5% activator, (**b**) partially enlarged below 200 °C, (**c**) 10% activator, (**d**) partially enlarged below 200 °C.

Figure 5 shows the thermal analysis results for the 1-day and 28-day samples for each mixing method. Figure 5a shows the TG/DTA analysis of 1-day and 28-day 5% activator samples. The weight loss seen at 50–200 °C was C-S-H gel [75,76]. The weight loss of the 28-day samples increased more than the at the 1-day weight loss rate. This means that C-S-H gel is formed due to the continuous hydration reaction of slag and FA with increasing age [77,78]. The main weight loss of the hydrated samples occurs between about 80 and 250 °C, which can be attributed to the sodium aluminosilicate hydrates [13].

Figure 5b shows an enlargement of 200 °C or less. The weight loss rate of Method-B was highest in both 1 day and 28 days. These means that Method-B has the effects of increasing the production of C-S-H gel compared to the other mixing methods.

Figure 5c shows the thermal analysis results of 10% activator samples. Similar to the 5% activator sample, the weight loss of the C-S-H gel at 50–200 °C is confirmed. The weight reduction rate of the 10% activator of Figure 5c was larger than that of Figure 5a of the 5% activator. This is because the hydration reaction of slag and FA was promoted as the dosage of activator increased. Figure 5d shows the weight reduction effect of each mixing method. As a result, Method-B showed the greatest weight reduction rate.

In the thermal analysis, Method-B showed the highest weight reduction rate, which means that the production of the hydration products C-S-H gel was increased. The increase in C-S-H gel affects the increase in compressive strength and the decrease in the diameter and volume of pores. The compressive strength of Method-B was the highest in the above-mentioned compressive strength results (see Figure 1 and Table 3). Method-B also showed that the most compact matrix was formed in the pore structure distribution (Figure 3), total porosity, and average pore diameter (Figure 4) in pore structure analysis.

In the XRD analysis of Figure 2, the change of the C-S-H gel peak according to the mixing method was insignificant. However, the results of the thermal analysis showed that there was a difference in the amount of hydration reactants produced by the mixing method. This difference in hydration reactants affects compressive strength and void structure. It was confirmed that the mechanical properties can be improved by forming a dense matrix by only changing the cycle (increase of mixing time, splitting of mixing water, and first injection of slag) without further adjustment of activator concentration and high temperature curing.

### 3.5. Microstructures

**Figure 6:**
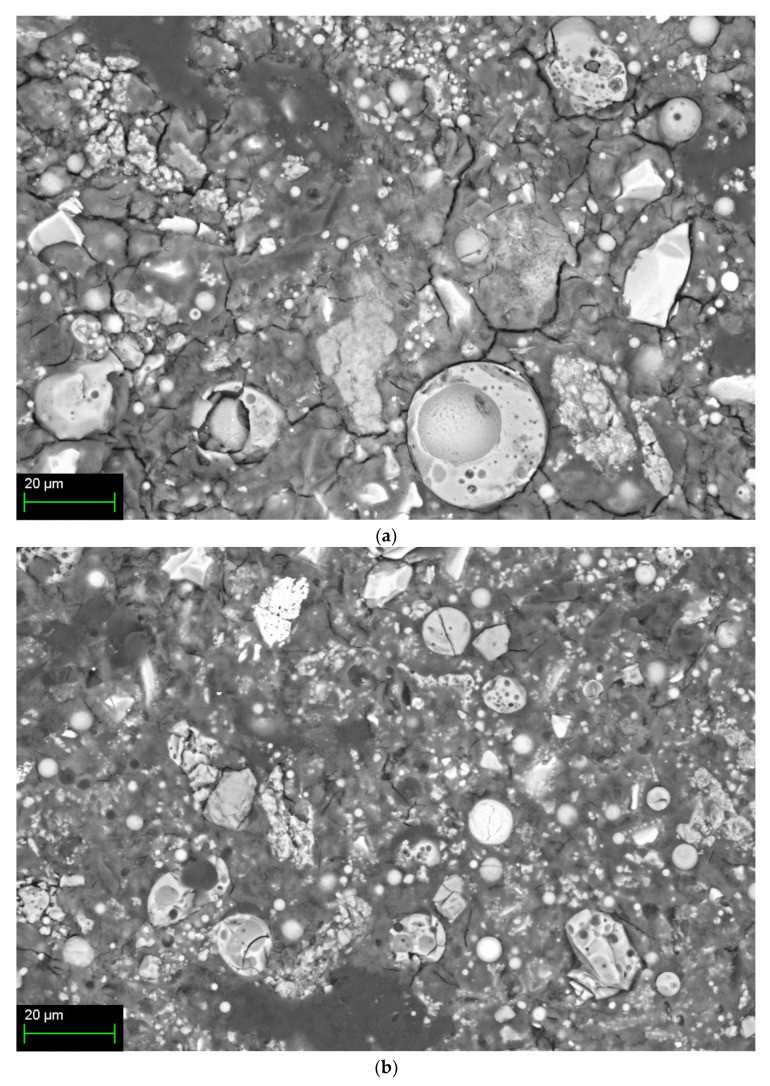

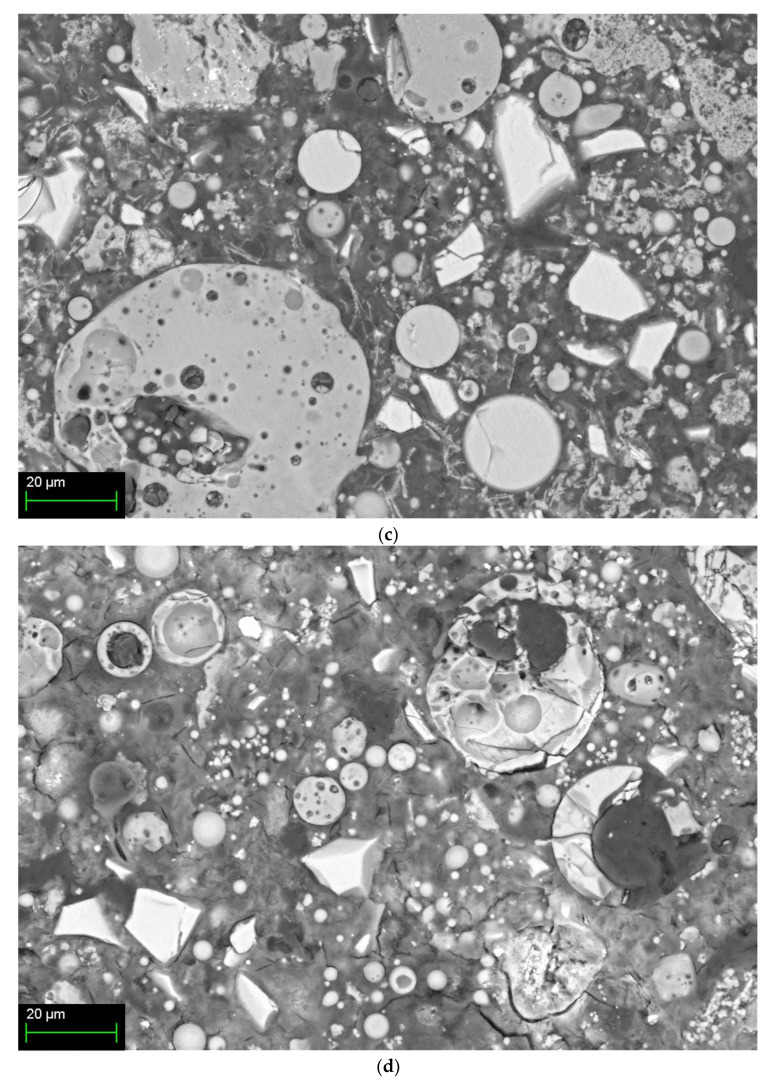
SEM/BSE image of 10% activator samples at 28-day for each mixing method: (**a**) Method-S, (**b**) Method-A, (**c**) Method-B, and (**d**) Method-C.

Figure 6 shows SEM images according to the mixing method of 10% activator samples at 28 days. Figure 6a shows the Method-S sample with unhydrated FA and partial-hydrated GGGFS particles with many cracks. Figure 6b is a Method-A sample with fewer cracks than Method-S. However, unhydrated FA and partially hydrated slag are still observed. The Method-B sample in Figure 6c shows no cracks and the most dense hydration products. A large number of partially hydrated slag particles are still observed. Figure 6d shows the Method-C sample, showing cracks, unhydrated FA, and partially hydrated slag particles, similar to Method-A.

The hydration reactivity of FA at room temperature is relatively low compared to slag, and many unhydrated FA particles are identified. The presence of such unhydrated FA particles can be presumed to result in less generation of FA hydration products such as N-A-S-H gel. The presence of low alkali concentrations and unhydrated FA at room temperature has already been reported in FA-based geopolymer studies [15,19,28,76].

Therefore, the acceleration of the hydration of slag forms a dense C-A-S-H gel [18,28]. In addition, the hydration reaction promotion of slag increases the elution of calcium ion from slag. The eluted calcium ions increase the compressive strength gradually over time through the process of accelerating FA hydration [18,31,38,79,80].

For slag–FA mixed binders, slag is said to form a high-density matrix hydration reaction product (C-(A)-S-H) [6,65,72]. In previous slag-FA studies, low FA reactivity at room temperature has been reported to form reaction products (N-A-S-H gel) with low strength and poor crystallinity due to unreacted FA particles [18]. The reaction products of the slag–FA mixed binder are N-A-S-H (geopolymer gel) from FA and C-(A)-S-H from slag. Previous studies have reported that these two hydration products are present at the same time [4,15,18,29,32,58,59]. However, some researchers have also noted the presence of C-N-S-H gel, which is a hybrid type of N-A-S-H and C-(A)-S-H gels [18]. In addition, some studies have found only C-(A)-S-H gels [34].

It is difficult to identify and distinguish amorphous N-A-S-H and C-A-S-H gels by XRD analysis [5]. Additionally, in the thermal analysis, the weight loss temperature ranges of the two types of hydration products overlap, making it difficult to distinguish clearly. For this reason, it was difficult to clearly distinguish between the two types of hydration products through the XRD analysis of Figure 2 and the TG/DTA analysis of Figure 5. Therefore, Ca/Si, Al/Si, and Na/Si ratios of hydration products were obtained by EDS analysis.

**Figure 7:**
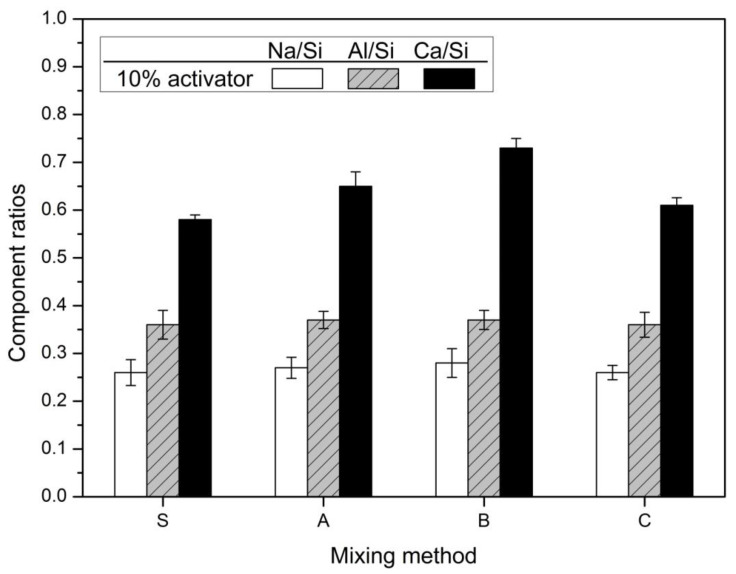
Component ratios of the hydration products.

Figure 7 depicted the results of the EDS analysis. The hydration reactants spots selected for analysis of EDS were five randomly selected spots. 

The Ca/Si and Al/Si ratios for the hydration products of slag-FA AAC have various ranges depending on the researchers. Marjanović et al. [36] reported that the Ca/Si ratio is 0.21–0.46 and the Al/Si ratio is 0.17–0.63 in the mixture with a GBFS-FA mixing ratio of 50:50. Abdalqaderet al. [44] mentioned that the Ca/Si ratio ranged from 0.6 to 1.0. In the results of Puertas and Fernández-Jiménez [35], the Ca/Si ratio of the main reaction product of the slag:FA = 50:50 mixture is ≤0.8, while Al/Ca ≤ 0.6 and Si/Al = 3.0. Activation of slag promotes calcium elution. As a result, the Ca/Si ratio increases. The Ca/Si ratio is consistent with this increasing trend as the substitution ratio of slag increases in previous studies on slag-FA alkali activated cements or geopolymers [36,40]. The EDS analysis of the slag-FA 50:50 mixture AAC indicated that the Si/Al ratios of the reaction products were 4.46 (C-S-H) and 4.02 (Ca-aluminosilicate) [7]. The Si/Al ratios of AAC with a 50:50 mixture of slag and Metakaolin in Buchwald et al. [81] were 2.8 (C-S-H) and 1.19 (aluminosilicate). In general, the range of Si/Al ratios of geopolymers varies (1 ≤ Si/Al ≤ 5) [82]. In previous studies, the Si/Al ratio of a high calcium geopolymer was found to be approximately 3.5 [83]. Depending on the constituent ratio of the binder, the ingredients of the raw materials, and the type and concentration of the activator, the proportions of the reactants vary. The study of Kumar et al. [15] was further supported by SEM-EDS studies that confirmed the formation of two types of reaction products: an aluminosilicate gel with Si/Al ≤ 2 and a C-S-H gel with Si/Al ≤ 2.5 and Ca/Si = 0.8.

The Ca/Si ratio of the hydration products of slag-FA AAC changes depending on the slag content. Previous studies of alkali-activated slag/fly ash paste reported that Ca/Si showed a linear correlation with compressive strength after 7 and 28 days of curing. The reported results indicate that the compressive strength increases with increasing Ca/Si [34]. 

In Figure 7, the 10% activator was 0.36, 0.37, 0.37, and 0.36. Al/Si ratios were almost similar regardless of the mixing method. When the hydration reactivity of FA is improved by the high concentration activator and the high curing temperature, the elution of aluminum increases and the Al/Si ratio increases. In addition, as the replacement rate of FA increases, the elution of aluminum increases and the Al/Si ratio increases [36,40]. It is believed that the mixing method has little effect on the hydration of FA. In contrast, Ca/Si shows the greatest change. In the 10% activator, it was 0.58, 0.65, 0.73, and 0.61. Ca/Si was the largest in Method-B, which is followed by Method-A, Method-C, and finally Method-S. The increase of Ca/Si value means an increase of elution of calcium ion by accelerating the hydration of slag. Thus, it is shown that Method-B is an effective method for promoting the hydration of slag. As a result, Method-B has the largest compressive strength in Figure 1 and was the most effective in decreasing the diameter of pores, as shown in Figure 3 and Figure 4.

Previous studies have reported that mortars with higher compressive strength form hydrate products with higher Na_2_O/Si_2_O ratios. This is due to the increase in the Na_2_O in the composition, which enhances the binding mechanism and properties of the geopolymers [40,46]. The Ca/Si ratio of the reaction products of GBFS-FA AAC increases as the content of slag increases [70]. The Ca/Si ratio of slag-FA AAC with 10–30% replacement of slag increases to 0.26–0.53, while the Na/Si ratio decreases to 0.37–0.26 [70]. Na is used to construct the reaction product of N-A-S-H (geopolymer gel) via the activation of FA [70].

In Figure 7, the Na/Si ratio was 0.26, 0.27, 0.28, and 0.26 for the 10% activator. Na/Si showed little change according to the mixing method. This is similar to the variation tendency of Al/Si according to the mixing method. The reason why the change of the values of Na/Si and Al/Si is insignificant regardless of the mixing method is that the FA hydration reactivity is low. This is because it is a high-alkaline environment for FA hydration reaction and a room-temperature curing environment rather than a high curing temperature condition. 

As a result, the weight loss of 50–200 °C can be judged by the influence of C-A-S-H gel rather than N-A-S-H gel in the thermal analysis result of Figure 5. Considering the slight change of Na/Si and Al/Si and the large range of change of Ca/Si determined by EDS analysis, it is reasonable to consider the weight loss by C-A-S-H gel, which is the hydration products of slag. Therefore, the mixing method increases the compressive strength due to the reduction of the pore diameter and the formation of the hydration products (C-A-S-H gel) by increasing the hydration reaction of slag. Method-B showed the greatest improvement in compressive strength due to these causes.

The EDS analysis results suggest that among the factors affecting the compressive strength change according to the mixing method, the hydration product of slag may be related. In particular, the mixing method considered in this study increases the activity of slag, while FA has a relatively low activity. This is due to the already known low hydration reactivity of FA at room temperature. Therefore, Method-B has the greatest effect of increasing the compressive strength by increasing the activation action of slag and improving the formation of hydration products.

## 4. Conclusions

An experiment was conducted to investigate the effect of the mixing method on the mechanical properties of alkali-activated cement (AAC) in which slag and FA were mixed in a ratio of 1:1. The basic mixing method (Method-S) is ASTM C305, and the method of adding the same mixing sequence and time (Method-A) was considered. In addition, in order to create a high-concentration alkaline environment, a method of first mixing half of the mixed water and mixing water of all activators, and then mixing the remaining half of the mixed water in the additional mixing step was also designed (Method-B and Method-C). The difference between Method-B and Method-C is that slag is first mixed and FA is mixed in an additional mixing step (Method-B) and vice versa (Method-C).

The mixing method in which half of the mixed water and the activator were mixed in the preceding step and the remaining half of the mixed water was mixed in the first step (Method-B and C) showed more improved mechanical properties. In particular, the method of mixing slag in the first mixing step and mixing FA in the second mixing step (Method-B) showed the highest compressive strength and dense pore structure.

The high alkali activator concentration promotes the hydration reaction of slag. In addition, the same mixing sequence and time added increases the contact area and time between the particles of slag and the activator, thereby showing a higher synergy effect. These results indicate that activating slag, which has a relatively higher activation reactivity than FA at room temperature, is an effective mixing method for improving the mechanical properties in slag-FA-based AAC.

The mixing method did not affect the change in the kind of hydration products. The addition of the mixing time and the method of mixing slag in the first mixing step confirmed that the formation of C-S-H gel, the main hydration product, was increased. In particular, the mixing method had a great influence on the pore structure. As the mixing time increased and the activation of slag was promoted, the total porosity and average pore diameter decreased. The addition of the same mixing time and sequence has the effect of homogeneous mixing of slag and FA particles and increasing the contact area and time with the activator. In addition, the method of mixing half of the mixed water and the total alkali activator in the first step and the other half of the mixed water in the second step accelerates the hydration reaction of slag in the first step. This results in high mechanical properties and dense matrix formation.

In addition, the higher the concentration of the activator, the denser the pore structure. In other words, 10% activator samples showed smaller total porosity, smaller average pore diameter, more hydration products, and higher compressive strength than 5% activator. This is because the relatively higher activator at room temperature promotes the hydration reaction of slag more than FA. Therefore, Method-S of the 10% activator formed the most dense matrix.

Therefore, it was confirmed that the mechanical properties can be improved by adjusting the mixing method without the use of process or material such as increase of activator concentration, use of additional supplementary cementitious materials, and high-temperature curing in alkali-activated slag-FA paste.

## Figures and Tables

**Table 1:** The properties of ground granulated blast furnace slag (slag) and fly ash (FA).

Properties	Level	Slag	FA
Chemical components (%)	SiO_2_	37.21	70.35
	Al_2_O_3_	9.07	15.82
	Fe_2_O_3_	0.54	3.08
	CaO	43.73	6.76
	MgO	3.69	0.81
	TiO_2_	0.45	-
	MnO	0.18	-
	SO_3_	3.52	0.78
	K_2_O	0.71	1.13
Physical properties	LOI (%)	1.84	1.09
	Blaine (m^2^/kg)	410	390
	Density (g/cm^3^)	2.86	2.24

**Table 2:** Mixing time and procedures.

Step	Method-S	Method-A	Method-B	Method-C
Preparation	Place the mixing water and activator in the bowl	Place the mixing water and activator in the bowl	Place half of the mixing water and activator in the bowl	Place half of the mixing water and activator in the bowl
First cycle	Add the slag and FA to the bowl (waiting for 30 s)	Add the slag and FA to the bowl (waiting for 30 s)	Add the slag to the bowl (waiting for 30 s)	Add the FA to the bowl (waiting for 30 s)
	Mixing at the slow speed (140 ± 5 r/min) for 30 s			
	Stop the mixer for 30 s. Scrape off all the paste that has collected on the side of the bowl.			
	Mixing at the medium speed (285 ± 10 r/min) for 30 s			
	Stop the mixer for 90 s.			
	Mixing at the medium speed (285 ± 10 r/min) for 60 s			
Second cycle		Stop for 30 s.	Add FA and half of the mixing water to the bowl and let it sit for 30 s.	Add slag and half of the mixing water to the bowl and let it sit for 30 s.
		Mixing at the slow speed (140 ± 5 r/min) for 30 s		
		Stop the mixer for 30 s. Scrape off all the paste that has collected on the side of the bowl.		
		Mixing at the medium speed (285 ± 10 r/min) for 30 s		
		Stop the mixer for 90 s.		
		Mixing at the medium speed (285 ± 10 r/min) for 60 s		

**Table 3:** Relative compressive strength increase rate for Method-S (%).

Age (Days)	5% Activator			10% Activator		
	Method-A	Method-B	Method-C	Method-A	Method-B	Method-C
1	107.5	115.5	103.8	119.7	133.2	117.3
3	113.6	126.4	111.6	123.2	129.6	116.9
7	120.7	128.8	114.5	126.8	140.6	112.4
28	116.2	127.8	110.1	123.1	132.1	113.2

## Data Availability

Data sharing is not applicable to this article.
